# High-throughput preparation methods of crude extract for robust cell-free protein synthesis

**DOI:** 10.1038/srep08663

**Published:** 2015-03-02

**Authors:** Yong-Chan Kwon, Michael C. Jewett

**Affiliations:** 1 Department of Chemical and Biological Engineering, Northwestern University, Evanston, IL 60208, USA; 2 Chemistry of Life Processes Institute, Northwestern University, Evanston, IL 60208, USA; 3 Robert H. Lurie Comprehensive Cancer Center, Medicine Northwestern University, Chicago, IL 60611, USA; 4 Institute of Bionanotechnology in Medicine Northwestern University, Chicago, IL 60611, USA

## Abstract

Crude extract based cell-free protein synthesis (CFPS) has emerged as a powerful technology platform for high-throughput protein production and genetic part characterization. Unfortunately, robust preparation of highly active extracts generally requires specialized and costly equipment and can be labor and time intensive. Moreover, cell lysis procedures can be hard to standardize, leading to different extract performance across laboratories. These challenges limit new entrants to the field and new applications, such as comprehensive genome engineering programs to improve extract performance. To address these challenges, we developed a generalizable and easily accessible high-throughput crude extract preparation method for CFPS based on sonication. To validate our approach, we investigated two *Escherichia coli* strains: BL21 Star™ (DE3) and a K12 MG1655 variant, achieving similar productivity (defined as CFPS yield in g/L) by varying only a few parameters. In addition, we observed identical productivity of cell extracts generated from culture volumes spanning three orders of magnitude (10 mL culture tubes to 10 L fermentation). We anticipate that our rapid and robust extract preparation method will speed-up screening of genomically engineered strains for CFPS applications, make possible highly active extracts from non-model organisms, and promote a more general use of CFPS in synthetic biology and biotechnology.

In recent years, a technical renaissance has revitalized *Escherichia coli*-based cell-free protein synthesis (CFPS) systems to match the increasing demands for simple, inexpensive, and efficient protein production^1^,^2^,^3^. Protein yields now exceed grams of protein per L reaction volume^4^, batch reaction duration has been extended to multiple hours^2^, and reaction scale has reached the 100-L milestone^5^ (a feat deemed impossible just over a decade ago).

The recent growth and interest in crude extract CFPS is motivated by the unique benefits provided by cell-free systems. From a biomanufacturing perspective, cell-free systems separate catalyst synthesis (cell growth) from catalyst utilization (protein production). This concept represents a significant departure from cell-based processes that rely on microscopic cellular ‘reactors' and allows the user to, in principle, harness all the metabolic resources that the cellular cytoplasm contains. From a prototyping perspective, barrier-free cell-free systems allow direct access to enzymes and reaction conditions. This dramatically increases the resolution at which the user can manipulate and sample the inner cellular environment. Indeed, such direct access, and the possibility to control the complex set of chemical reactions of native living systems, makes CFPS systems well-suited for understanding and developing representative biosynthetic processes. There is a high degree of flexibility to experimentally isolate biochemical processes from the confounding background of biological networks present in living organisms. Together, these advantages have inspired the use of crude extract based CFPS for basic research and applied biotechnology, including: synthesis of therapeutics^6^,^7^,^8^, evolution of proteins^9^,^10^, genetically encoding non-standard amino acids^11^,^12^,^13^,^14^,^15^,^16^, synthesis of virus like particles^17^,^18^,^19^, production of entire bacteriophages from genomic DNA^20^, production of disulfide bonded proteins^21^,^22^,^23^, synthesis and screening of enzymes^24^,^25^,^26^,^27^,^28^, production of membrane proteins^29^,^30^,^31^, construction of synthetic ribosomes^32^,^33^,^34^, high-throughput protein production and genetic part characterization^35^,^36^, and paper based diagnostics^37^, among others. As an alternative approach, cell-free translation using purified elements, or the PURE system initially developed by Ueda and colleagues, offers some important technological capabilities^38^,^39^,^40^,^41^. However, since crude cell lysates offer significantly lower system catalyst costs, we focus on them.

To produce proteins, *E. coli* S30 extract-based CFPS systems harness an ensemble of catalytic components from crude lysates of cells. Cellular lysates contain active enzymes, as well as helper factors for transcription, translation and protein folding. Activated catalysts within the cell lysate act, in coordination, as a working chemical factory to synthesize and fold desired protein products upon incubation with essential substrates (*e.g.*, amino acids, nucleotides, DNA or mRNA, energy substrates, cofactors, and salts). A variety of cell extract preparation methods have been developed for CFPS. In general, cells are harvested during mid-exponential growth of a low-density fermentation and then processed by high-pressure lysis (~20,000 *psig*), two 30,000 × *g* centrifugations (to remove cell wall fragments and genomic DNA), a run-off reaction (incubation of the clarified extract but no exogenous mRNA or DNA), and dialysis (to provide a suitable storage buffer)^42^,^43^ (Figure 1). Although first described in 1963^44^, many minor changes have been made to the original extract preparation procedures to streamline the process, improve productivity, and reduce background expression (Figure 1)^45^,^46^.

However, the cell extract preparation procedure generally remains a time and labor-intensive process. In addition, extract preparation procedures have been difficult to standardize, leading to different extract performance within and across laboratories. Furthermore, since most extract preparation procedures involve a large volume of cell culture (>1 L) and costly high pressure disruption equipment to lyse cells (*e.g.*, French Press or impinge homogenizer), obtaining and utilizing a large number of cell extracts from different source strains is not yet routine. This may be important for studying the systems effect of numerous genomic modifications to identify negative and positive effectors of protein synthesis for basic and applied research.

To address some of the aforementioned issues, Shrestha *et al.* recently streamlined a CFPS extract preparation method using equipment common to most biotechnology laboratories (Figure 1). Specifically, they used shake flask growth and sonication to reduce cost and variability, eliminating the need for specialized and expensive growth and lysis equipment^47^. This was a significant advance. However, their study was insufficient to provide general guidelines for optimal conditions needed to (i) lyse different cell suspension volumes, (ii) use different *E. coli* strains, and (iii) prepare lysates in a high-throughput manner from disposable mini-culture tubes.

Here, we sought to build off the Shrestha *et al.* method to develop a rapid, robust, and high-throughput extract preparation procedure using sonication that could reduce the time to make numerous extracts to ~1 day per 100 extracts, and do so consistently. The goal was to be able to generate ~100 μL of extract (which is sufficient for ~25 CFPS prototyping reactions at the 15 μL scale). We expected this could be achieved at the 10 mL culture volume scale. Our technology development study involved three steps. First, we explored the effect of a numerous sonication variables (energy input, sonication burst time, cooling time, etc.) on extract performance in CFPS. Second, after fixing the aforementioned sonication parameters, we mapped CFPS yields as a function of sonication energy input versus cell suspension volume. Specifically, we generated 144 cellular extracts of a commercially available *E. coli* strain BL21 Star™ (DE3) and a genomically recoded strain from strain K12 MG1655. Looking at multiple strains enabled us to assess strain differences on lysis and extract performance and ascertain the potential for our protocol to be general. Third, concerning the robustness of our method to make active crude cell extracts, we characterized our approach across a wide range of culture volumes (from 10 mL culture tubes to lab scale fermentation (10 L)). We additionally showed the ability to prepare lysates across multiple extract volumes (100 μL to 30 mL) with identical CFPS performance. In sum, our work yielded an efficient and accurate sonication method that has the potential to be applied to any strain of *E. coli.* Furthermore, it cataloged the impact of optimizing multiple extract preparation procedures together, which is expected to provide utility for developing CFPS systems from many prokaryotic species (*e.g., Streptomyces, Pseudomonas, etc.*).

## Results and Discussion

### Preparation of crude cell extract by sonication

**Figure 2:**
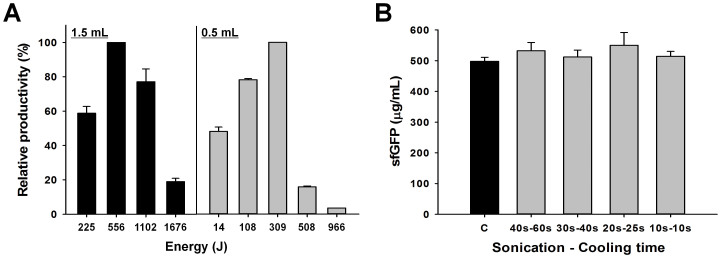
Sonication energy input is a critical parameter for preparation of highly active crude cell extracts. (A) Relative protein synthesis activities from crude extracts prepared with different sonication energy input (in Joules) in 1.5 mL and 0.5 mL cell suspensions. Numbers on the x-axis are total input energy. Fluorescence of sfGFP was measured following a 4 hour CFPS reaction. (B) Protein synthesis activities from crude extracts prepared with different sonication-cooling intervals. For each extract, the same sonication energy input value (556 J) and 1.5 mL cell suspension was used. Energy accumulation was monitored during lysis and then sonication was stopped when the total energy input is reached to 556 J. “C,” positive control, cell extract prepared by 10 L fermentation for culture and homogenization to lyse cells. sfGFP reported was based on the amount of active (fluorescent) sfGFP following a 4 hour CFPS reaction. 95% of the total sfGFP synthesized was active. Values represent averages and error bars represent standard deviation for at least 3 independent experiments.

We began our investigation by assessing the impact of total sonication energy input on cell lysis and crude extract performance, which we defined by the total amount of protein produced. To do this, we first cultured 1 L of BL21 Star™ (DE3) cells in 2.5 L baffled tunair shake flasks, which showed similar growth rate as compared to 10 L fermentation (Supplementary Table S1). Then, thawed cell suspensions of different volumes were lysed using multiple sonication input energies (expressed in Joules), with a constant sonication burst - cooling (at 4°C) cycle of 45 s–60 s (s: seconds). Cell resuspensions were not stirred during sonication. Next, crude S30 extracts were prepared from these cell lysates. Subsequently, 15 μL batch-mode transcription and translation (TX-TL) reactions were performed for 4 hours at 37°C to determine the amount of superfolder green fluorescent protein (sfGFP) synthesized. These experiments showed that extract performance depended greatly on the sonication energy input (Figure 2A). Our experiments suggest that if too little sonication energy is used, not enough cells are burst, which results in crude extracts with lower total *E. coli* protein concentration (Supplementary Figure S1). On the other hand, higher sonication energy levels sufficiently lyse the cellular suspension, but can also deactivate the catalysts present in the extract. Most likely, this is due to the heat shock introduced after many sonication-cooling cycles. Consistent with these design rules, we observed different optimal total sonication energy inputs in relation to different cell suspension volumes (556 J for 1.5 mL and 309 J for 0.5 mL).

We hypothesized that a given cell suspension volume with the same total sonication energy input would exhibit the same protein biosynthesis performance if heat shock was avoided. In other words, a consistent amount of energy is needed to lyse a defined amount of cells. We therefore investigated sonication-cooling intervals for preparing small volumes of cell extract, being careful to avoid any detrimental effect on protein synthesis due to an excessive flow of heat during cell disruption. In particular, to achieve 556 J of energy input for 1.5 mL cell suspension, we monitored four different cycles of the following sonication-cooling conditions; 40 s–60 s, 30 s–40 s, 20 s–25 s, and 10 s–10 s. Consistent with our hypothesis, as long as the total energy input per cell suspension volume was constant, we observed no significant difference in extract activity under different sonication burst periods and 4°C cooling intervals (Figure 2B). These data suggest that highly active extracts can be produced with minimum sample heating by identifying an optimum energy input per cell suspension volume and then use of a short sonication-cooling cycle (10 s–10 s).

### Optimization of cell extract preparation procedure with different bacteria strains

After we defined a reproducible sonication cell lysis strategy to generate highly active extracts, we decided to perform a series of optimization experiments to identify conditions for robust and consistent extract preparation. We chose to explore the impact of several pre- and post-lysis steps, such as: culture harvest time, the ratio between wet cell pellet weight and buffer A, centrifugation speed, and run-off reaction time. In addition, we chose to study two strains (BL21 Star™ (DE3), as above, and also a genomically recoded derivative of K12 MG1655 (strain C495^48^)). This allowed us to see if our protocol was robust to both B- and K- strains of *E. coli*.

**Figure 3:**
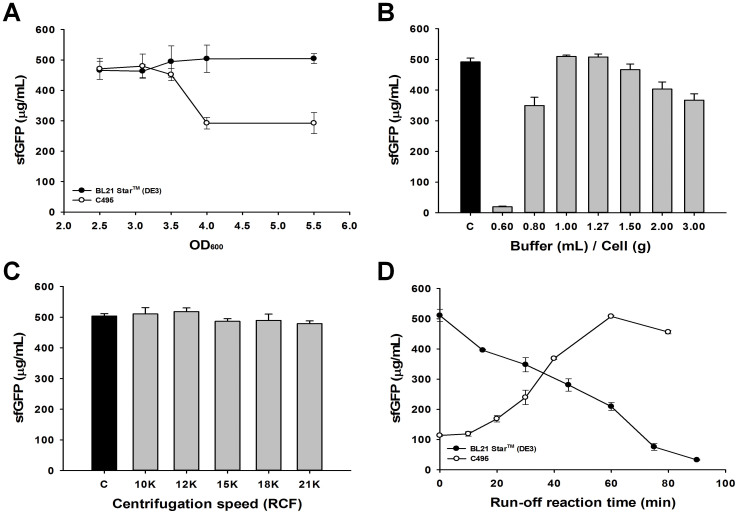
Optimization of cell extract preparation procedure when using sonication for cell lysis. (A) CFPS activity of extracts prepared from cells grown in 1 L of 2 × YTPG media and harvested at different optical densities. Extracts were prepared by sonicating 1.5 mL of cell suspension with 556 J. (B) CFPS activity of cell extracts prepared using varying ratios of wet cell pellet to buffer A for resuspension. Ratios are given in terms of wet cell weight in g per mL of buffer A. (C) CFPS activity of cell extracts prepared using varying centrifugation speeds for the first spin. The cell extracts were prepared without the run-off reaction and no second centrifugation. (D) The impact of run-off reaction times for preparation of active crude cell extracts. The run-off reaction was carried out at 37°C with shaking (250 rpm). In A and D, filled circles and open circles represent the CFPS activities of BL21 Star™ (DE3) and C495 cell extracts, respectively. In B and C, extracts were generated from BL21 Star™ (DE3). In B and C, black bars show the activity of a positive control cell extract prepared by 10 L fermentation for culture and impinge homogenization to lyse cells. sfGFP reported was based on the amount of active (fluorescent) sfGFP following a 4 hour CFPS reaction. Values represent averages and error bars represent standard deviation for at least 3 independent experiments.

The composition of the cellular machinery at the time of harvest directly affects the CFPS potential of the crude extract. In *E. coli* CFPS systems, it has been shown that exponential growth phase is the best time to harvest the cells during culture because the translation machinery is most active^49^,^50^. However, cells harvested at a higher optical density result in a larger cell mass. In turn, leading to an increased quantity of total crude extract prepared per fermentation. Accordingly, we grew *E. coli* cells to 2.5, 3.1, 3.5, 4.0, and 5.5 OD_600_, which spanned a range of mid to late exponential growth in our culture media, and prepared individual batches of crude extract originating from each of these cultivations. Specifically, we prepared a set of 1.5 mL crude cell extracts with 556 J of energy input using sonication-cooling cycles of 10 s–10 s. Overall CFPS activity from BL21 Star™ (DE3) strain lysates was fairly consistent at any OD_600_ points during exponential growth phase (OD_600_ 2.5 ~ 5.5) (Figure 3A). In contrast, strain C495 showed that the most active extracts were obtained from culture harvested at mid-exponential phase (468 ± 14 μg mL^−1^ for OD_600_ 2.5–3.5 compared to 292 ± 2 μg mL^−1^ for OD_600_ of 4.0–5.5) (Figure 3A). While the reason for the significant impact of harvest OD on strain C495 remains unknown, the 40% reduction in activity might be related to the slower growth of these cells as compared to BL21 Star™ (DE3). C495 has a 30% increase in doubling time as compared to BL21 Star™ (DE3) (Supplementary Table S1). We went on to show that CFPS activities from both strain lysates were reduced if the cells were harvested in stationary phase (Supplementary Figure S2). Taken together, our data highlight the importance of cell harvest as a key parameter for optimization and batch-to-batch variability. Next, we compared the ratio of cell pellet to buffer suspension for preparing highly active cell extracts by sonication cell lysis. Ratios of 1:1 and 1:1.27 (wet cell pellet weight in g to volume of buffer in mL) showed the highest extract activity after lysis (Figure 3B).

Beyond assessing the impact of pre-lysis steps, we also investigated post-lysis extract preparation steps. Specifically, we evaluated the impact of centrifugation speed on the activity of prepared extracts, as this represents a critical step in streamlining crude extract preparation. The centrifugation speed was not critically influential on the activity of cell extract for CFPS. We observed that speeds over 10,000 RCF (4°C for 10 min) for the first centrifugation provided no significant difference in activity among extract samples (Figure 3C), noting that these speeds were sufficient to remove all un-lysed cells (see below).

We next evaluated the effect of the run-off reaction, which consists of supernatant incubation at 37°C with 250 rpm agitation for a specified time after the first centrifugation. Strikingly, lysate performance in CFPS by the two different *E. coli* strains showed very different activity. While extracts from strain BL21 Star™ (DE3) exhibited a gradual decrease of CFPS activity with extended run-off reaction times, those from strain C495 showed the run-off reaction time as an essential parameter to enhance CFPS performance (Figure 3D). Notably, the run-off reaction step was followed by a second centrifugation (10,000 RCF at 4°C for 10 min), which we found to be important for high CFPS activity when using the run-off reaction. In addition, particular attention was taken to not transfer any cellular debris into the final lysate following the run-off reaction in order to maintain active cellular lysates. By avoiding cellular debris, we were also able to produce lysates without remaining *E. coli* cells. This requires care in separating the supernatant from the cell pellet at the expense of not collecting all of the lysate.

We next tested the possibility of overexpressing T7 RNA polymerase within the source strain for CFPS as previously reported^45^. Specifically, we compared CFPS activities between T7 RNA polymerase overexpressed BL21 Star™ (DE3) cell extract and purified T7 RNA polymerase that was exogenously supplied to extracts from an uninduced BL21 Star™ (DE3) strain (Supplementary Figure S3). Although T7 RNA polymerase overexpressed extracts showed slightly higher CFPS activity, purified T7 RNA polymerase was used for the experiments below so that we could compare results from both *E. coli* strains: BL21 Star™ (DE3) and C495. This is because the C495 strain lacks genomically integrated T7 RNA polymerase.

### Fine mapping of sonication energy input versus cell suspension volume

**Figure 4:**
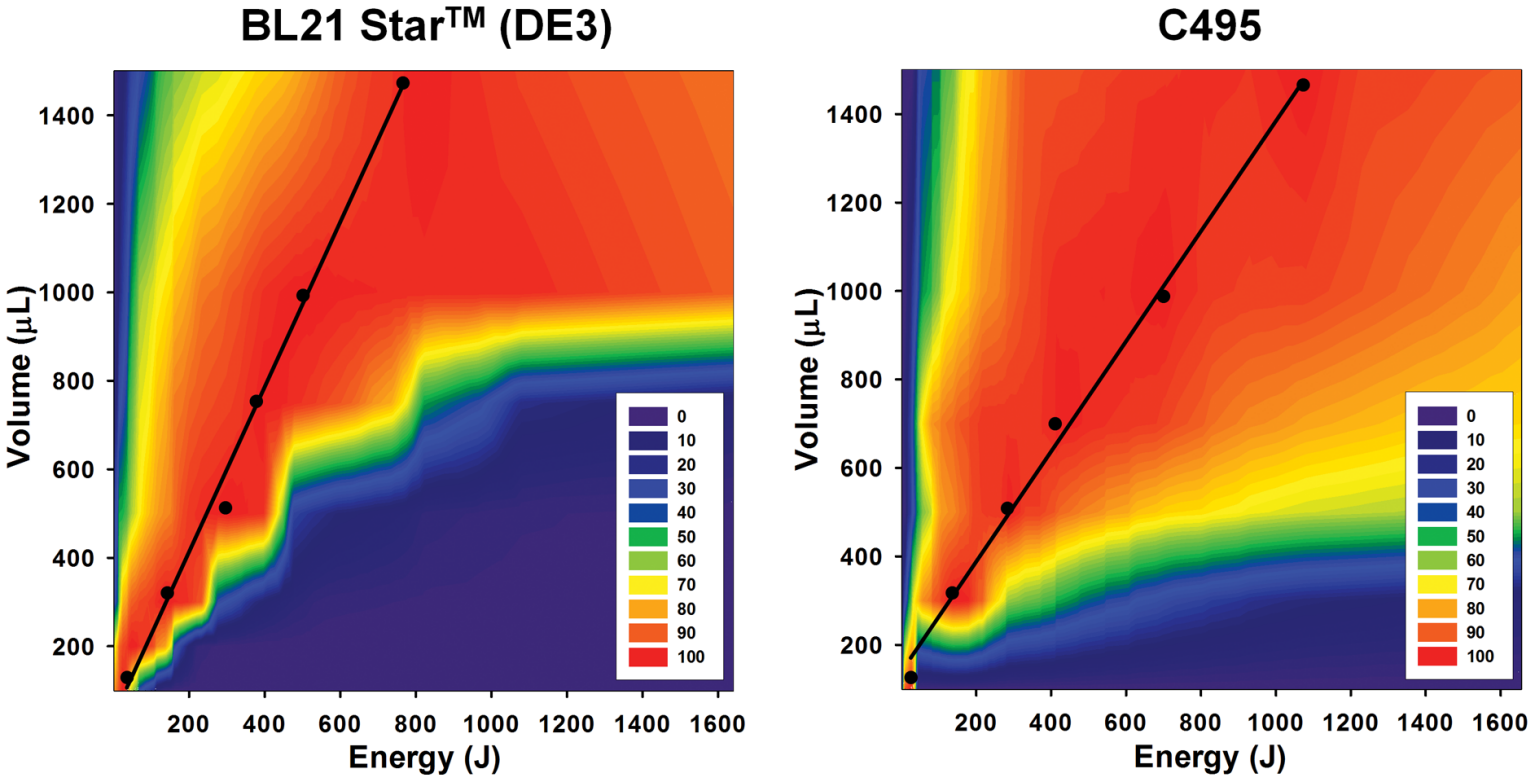
Mapping CFPS yields across the landscape of total sonication energy input and cell suspension volume demonstrates the ability to generate highly active extracts. CFPS activity was mapped for two *E. coli* strains, showing a linear trend for the highest CFPS activity. The color code represents relative CFPS activities (100% (red) to 0% (purple)) for each designated energy and volume. Linear trends for total energy inputs covering the highest CFPS activity are computed as follows: Equation for BL21 Star™ (DE3): [Energy] = [Volume(μL)-33.6]·1.8^−1^, Equation for C495: [Energy] = [Volume(μL) − 112.7]·1.3^−1^.

After we evaluated all the steps in our sonication based cell extract preparation, we subsequently determined the ideal balance between sonication energy input and cell suspension volume. This represents a step forward compared to previous studies where cell suspension volume was kept constant^47^. Our goal was to reduce the overall volume for each step of the preparation procedure, from cell growth to crude cell extract preparation, such that one might imagine using culture tubes to prepare source cells for tens to hundreds of mutants and their cell lysates in high-throughput. The key point was to identify a correlation between sonication energy input and volume, such that we can predict an optimal parameter configuration to generate highly active extract from different cell strains and suspension volumes. To do this, we mapped the sonication energy input versus cell suspension volume by generating 144 extracts for the BL21 Star™ (DE3) and C495 strains. For each strain, we observed maximal energy inputs for each cell suspension volume when preparing highly active crude cell extract (Figure 4). For illustration purposes, 5 and 6 short sonication-cooling cycles (10 s–10 s) were needed at the optimum energy input for 0.5 mL cell suspensions from the BL21 Star™ (DE3) and C495 strains, respectively. While small volumes of cell suspension were very sensitive to energy input (*i.e.*, the maximum values were tightly banded in Figure 4), large volumes of cell extracts showed a relatively high tolerance against high-energy input (Supplementary Figure S4). Thus, to obtain highly active crude cell extracts from the small volume, a precise titration of energy input must be imposed.

**Figure 5:**
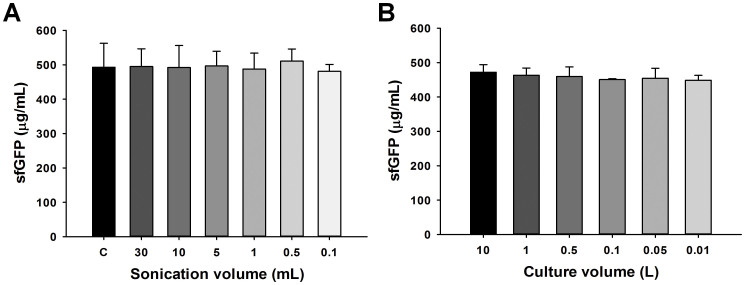
The impact of sonication volume and culture volume on the preparation of robust and high performing crude cell extracts for CFPS. (A) The impact of sonication volume on CFPS activity. All sonication volume variants were prepared from the same 50 mL of cell and buffer mixture. Energy was delivered until it reached to the optimal energy input per the desired cell suspension volume. Optimal energy input was calculated from energy and volume correlation map (Figure 4 and Table 1). “C,” positive control, cell extract prepared by 10 L fermentation for culture and homogenization to lyse cells. (B) The impact of culture volume on CFPS activity. sfGFP reported was based on the amount of active (fluorescent) sfGFP following a 4 hour CFPS reaction. Values represent averages and error bars represent standard deviation for at least 3 independent experiments.

Notably, the two strains also showed different maximization profiles. The C495 strain has a broader tolerance of sonication energy inputs per cell suspension volume when compared to BL21 Star™ (DE3). Even though both *E. coli* strains showed different patterns, the correlation of energy and volume exhibited a linear trend in both strains (Figure 4). In accordance with the linear trend, we were able to predict the optimal amount of energy input from user-designated volume for highly active extract. For instance, in follow-up experiments, we built a calibration line using only a few points to choose parameters for efficient extract preparation. As shown in Table 1, except for the smallest volumes (100 μL), predicted energy input for the highest activity of cell extract is well fitted to the experimental data (globally found to be within 5.2 ± 4.4%). Next, we showed the ability to prepare lysates across multiple extract volumes (100 μL to 30 mL) with identical CFPS performance (Figure 5A). Overall, these data highlight that our approach is robust and predictive. Moreover, it outlines a detailed, generalized method to characterize and prepare extracts from multiple strains using sonication-based lysis.

### High-throughput cell extract preparation

Upon demonstration of a generalizable approach for maximizing extract quality by sonication cell lysis, we then examined the ability to make active crude cell extracts from small to large culture volumes (10 mL culture tubes to 10 L lab scale fermentation). This is important because our standardized protocol for small culture volumes makes CFPS an accessible tool to many researchers with inexpensive equipment. While our method does require a sonicator with the ability to measure energy input in Joules, we have identified many manufacturers are available to meet this requirement. In addition, our method is simple and can be executed in high-throughput fashion for high-throughput protein expression, testing bacterial strains harboring different genomic mutations, and perhaps for rapid design-build-test cycles in the laboratory to assess genetic constructs or circuits (*i.e.*, biomolecular breadboarding)^51^.

To test small to large culture sizes, we cultured BL21 Star™ (DE3) cells in multiple culture volumes (10 L, 1 L, 500 mL, 100 mL, 50 mL, and 10 mL) and prepared cell extracts from this strain exploiting the linear correlation found in the heat map (Figure 4). We used a steam-in-place fermentor for 10 L culture, 2.5 L baffled tunair shake flasks for large culture (1 L and 500 mL) and 300 mL baffled tunair shake flasks for small cultures (100 mL and 50 mL). For the 10 mL culture, we grew the cells in standard glass culture tubes. In order to ensure consistency across different cultivation conditions, we carefully monitored cell growth rates and harvested all cells at an OD_600_ of 3. Cells cultured in the 10 L fermentor or shake flasks showed similar growth rates within 13% variation. Whereas, the growth rate of the 10 mL culture in the culture tube was 44% slower (Supplementary Table S1). We hypothesized that the slower growth rate in the culture tube was due to inefficient mixing and reduced oxygen transfer. Given the slower growth rate, we might have expected that the cell extract from the 10 mL culture tube would have had lower activity. Instead, all cell extracts from different culture volumes have the same CFPS activity (Figure 5B). These data suggest that there may be a common mechanism limiting CFPS. One possible explanation is that dilution of translation factors is a limitation. Underwood, Swartz, and Puglisi have showed this limitation previously^52^. At longer batch reaction times, small energy molecules and phosphate accumulation in the PEP based energy regeneration system used here are expected to limit CFPS yields, as reported previously^4^,^53^,^54^,^55^.

The results described above are important due to the practical implications that an extract preparation from 10 mL culture will provide, in particular, by reducing the time and effort for making numerous different extracts. The wet weight of cells from a 10 mL culture tube was about 0.07 g at harvest OD_600_ 3.0, which was a sufficient quantity to prepare a 100 μL cell suspension for sonication. Only a few seconds (~5 s) and a sonication energy input of about 25 J were necessary to efficiently lyse 100 μL and generate a highly efficient cell extract for CFPS. While the BL21 Star™ (DE3) cells demonstrated similar protein synthesis yields from 10 mL to 10 L cultures, extracts from strain C495 did not perform well at the 10 mL scale for unknown reasons (data not shown).

## Conclusions

In summary, we developed a rapid, robust, and high-throughput sonication based cell extract preparation method. We identified that sonication energy input is a critical parameter that needs to be modulated to achieve efficient and highly active CFPS. We also defined a systematic approach to develop sonication based cell lysis procedures adaptable for multiple bacterial strains. By detailed mapping of sonication energy input versus cell volume suspension, we discovered a linear correlation in the fitness landscape that we found useful to extend our approach to a variety of cell suspensions (from the 100 μL to the 30 mL scale). In addition, we showed a scaling factor of ~10^3^ for different cell culture volumes (*i.e.*, CFPS yields were similar from source strains grown from the 10 mL to the 10 L scale), reducing the cell growth volume to as low as 10 mL for the BL21 Star™ (DE3) strain. We successfully minimized culture volume without compromising CFPS performance. Therefore, our method can significantly reduce the time for generating highly active extracts, which have previously been based on expensive and laborious equipment for culture and cell lysis. The sonication based extract generation reported here can accelerate the time for generating 100 extracts to potentially ~1 day (including culture time) as compared to about 250 days with our previous approach that required a 10 L fermentor and high pressure impinge homogenizer (Supplementary Figure S5). Finally, we note that lysates prepared by sonication can produce high protein titers in CFPS. While CFPS reactions reported throughout the paper were carried out for only 4 hours for rapid prototyping and consistency, Supplementary Figure S6 shows that protein yields are ~1 mg/mL from sonicated lysates when the reactions are allowed to go to completion (20 h). The cell extract preparation method demonstrated in this study not only provides a readily available, consistent approach for preparing *E. coli* based crude cell extract, but also an opportunity for fast characterization of genomically engineered *E. coli* extracts in CFPS at high yields. We believe that this work will help to overcome current limitations in preparing cell extracts for high-throughput processes. We also anticipate that this method will greatly increase the ‘design-build-test' cycles for a wide variety of synthetic biology studies.

## Methods

### Materials

*E. coli* BL21 Star™ (DE3) strain was purchased from Life Technologies (Grand Island, NY). Strain C495 (also reported as MCJ.495) is a genomically engineered variant of K12 MG1655 described previously^48^. *E. coli* total tRNA mixture (from strain MRE600) was purchased from Roche Applied Science (Indianapolis, IN). ATP, GTP, CTP, UTP, Phosphoenolpyruvate, 20 amino acids and other materials were purchased from Sigma (St. Louis, MO) without further purification. sfGFP was cloned into pY71 vector using NdeI and SalI restriction site and used as model protein in this study. T7 RNA Polymerase was obtained by affinity tag purification^56^.

### Preparation of cell extracts

*E. coli* BL21 Star™ (DE3) and C495 cells were grown in 2 × YTPG media at 34°C. We grew the strains at 34°C such that we could compare the strains. The MG1655-based C495 strain harbors a temperature sensitive λ-Red recombinase that requires growth at a lower temperature. Antibiotics were not used during cell growth. The cells were grown in a BIOSTAT C-plus fermentor (Sartorious AG, Goettingen, Germany) at 600 rpm for 10 L large-scale cultures. For preparing 1 L scale cultures, cells were grown in 2.5 L baffled tunair shake flasks (IBI Scientific, Peosta, IA) in a 34°C incubator with vigorous shaking at 250 rpm. For smaller scale cultivation (50 to 100 mL culture), 300 mL baffled tunair flasks were used. Unless otherwise specified in the text, the cultured cells were monitored by spectrophotometry (Genesys 10S UV-Vis, Thermo Fisher Scientific, Waltham, MA) until OD_600_ reached at 3.0. The maximum OD_600_ for BL21 Star™ (DE3) and C495 strains measured after 22 h culture that reached saturation were ~10 and ~8, respectively. The cells were harvested in the middle of exponential growth phase by centrifuging at 5000 RCF at 4°C for 15 min and were washed three times with cold Buffer A. Buffer A contained 10 mM Tris-acetate (pH 8.2), 14 mM magnesium acetate, 60 mM potassium glutamate, and 2 mM dithiothreitol. After final wash and centrifugation, the pelleted wet cells were weighed, flash frozen in liquid nitrogen, and stored at −80°C. The thawed cells were suspended in 1 mL of Buffer A per 1 g of wet cell mass. For obtaining standard “control” large-scale cultured cell extract, suspended cells were disrupted by EmulsiFlex-C3 homogenizer (Avestin, Ottawa, Canada) with single pass at a variable pressure of 20,000 to 25,000 psig. In order to remove cell debris and insoluble components in the cell lysate, the lysate was centrifuged twice at 30,000 RCF at 4°C for 30 min. The supernatant was then incubated at 37°C for 60 min with gentle shaking (250 rpm) and centrifuged at 15,000 RCF at 4°C for 15 min. In order to lyse cells by sonication, thawed and suspended cells were transferred into 1.5 mL microtube and placed in an ice-water bath to minimize heat damage during sonication. The cells were lysed using a Q125 Sonicator (Qsonica, Newtown, CT) with 3.175 mm diameter probe at frequency of 20 kHz and 50% of amplitude. The input energy (Joules) was monitored and recorded during sonication. The lysate was then centrifuged once at 12,000 RCF at 4°C for 10 min. The run-off reaction (37°C at 250 rpm) and second centrifugation (10,000 RCF at 4°C for 10 min) were performed for strain C495. However, in the case of strain BL21 Star™ (DE3), the run-off reaction and second centrifugation step were not needed. The total amount of protein in cell extract was quantified by Bradford assay. All extracts contained 40.7 ± 2.6 mg/mL of total *E. coli* proteins. All of prepared cell extract was flash frozen in liquid nitrogen and stored at −80°C until use.

### Cell-free protein synthesis

The CFPS reactions were carried out in a 1.5 mL microtube in the incubator. The standard reaction mixture for CFPS consists of the following components in a final volume of 15 μL: 1.2 mM ATP; 0.85 mM each of GTP, UTP, and CTP; 34.0 μg mL^−1^ L-5-formyl-5, 6, 7, 8-tetrahydrofolic acid (folinic acid); 170.0 μg mL^−1^ of *E. coli* tRNA mixture; 130 mM potassium glutamate; 10 mM ammonium glutamate; 12 mM magnesium glutamate; 2 mM each of 20 amino acids; 10 μM of L-[^14^C(U)]-leucine (11.1 GBq mmol^−1^, PerkinElmer, Waltham, MA); 0.33 mM nicotinamide adenine dinucleotide (NAD); 0.27 mM coenzyme-A (CoA); 1.5 mM spermidine; 1 mM putrescine; 4 mM sodium oxalate; 33 mM phosphoenolpyruvate (PEP); 13.3 μg mL^−1^ plasmid; 100 μg mL^−1^ T7 RNA polymerase, and 27% v/v of cell extract. The CFPS reactions were carried out at 37°C for 4 hours.

### Quantitative analysis of synthesized protein

The amounts of the cell-free synthesized protein were quantified from the TCA-insoluble radioactivity calculation using a liquid scintillation counter (MicroBeta2, PerkinElmer, Waltham, MA) as described elsewhere^55^,^57^. Fluorescence of active sfGFP was measured by multi well plate fluorometer (Synergy2, BioTek, Winwooski, VT). 2 μL of cell-free synthesized sfGFP was mixed with 48 μL of purified water and placed in a flat bottom of 96-well half area black plate (Corning Incorporated, Corning, NY). Excitation and emission wavelength for fluorescence of sfGFP were 485 and 528 nm, respectively. Total and cell-free synthesized protein was determined by Coomassie-blue staining analysis on a 4–12% SDS-PAGE gel (Life Technology, Grand Island, NY).

## Author Contributions

Y.-C.K. and M.C.J. designed the experiments, Y.-C.K. carried out the experiments and analyzed data, Y.-C.K. and M.C.J. wrote and approved the manuscript.

## Supplementary Material

Supplementary Information Supplementary Information

## Figures and Tables

**Figure 1:**
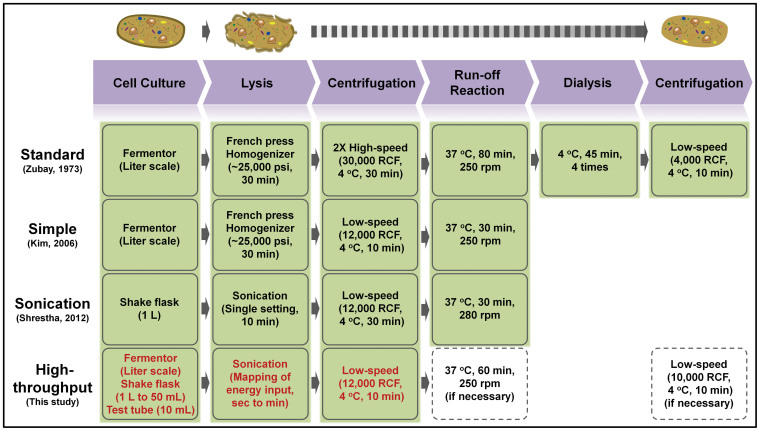
Cartoon highlighting a variety of representative methods for *E. coli* crude extract preparation.

**Table 1:** Total sonication energy input versus cell suspension volume for maximal CFPS productivity

Strain	Cell suspension volume (μL)	Sonication energy (J)			
		Predicted energy input for >95% productivity	Predicted energy input for 100% productivity	Observed energy input for 100% productivity	Difference (%)^a^
BL21 Star™ (DE3)	100	24 ± 11	37	24	54.2
	300	143 ± 39	150	140	7.1
	500	299 ± 71	263	309	15.0
	750	426 ± 72	404	398	1.5
	1000	639 ± 206	545	532	2.4
	1500	798 ± 105	826	822	0.5
K12 MG1655 C495	100	23 ± 6	−10	26	138.0
	300	146 ± 32	145	138	5.0
	500	290 ± 38	300	285	5.1
	700	417 ± 143	454	414	9.7
	1000	613 ± 151	686	705	3.0
	1500	940 ± 230	1073	1080	3.0

(a)Energy differences between predicted and observed 100% productivity.
