# Supplementary material for: High-throughput preparation methods of crude extract for robust cell-free protein synthesis
Source: Sci Rep. 2015 Mar 2;5:8663. doi: 10.1038/srep08663 (PMC4345344; doi:10.1038/srep08663)
Supplement: Supplementary Information [file srep08663-s1.pdf]

Supplementary Information

High-throughput preparation methods of crude extract for robust cell-free protein synthesis

Yong-Chan Kwon[1,2] and Michael C. Jewett[1,2,3,4*]

[1]Department of Chemical and Biological Engineering, [2]Chemistry of Life Processes Institute, Northwestern University, Evanston, IL 60208, USA

[3]Robert H. Lurie Comprehensive Cancer Center, [4]Institute of Bionanotechnology in Medicine Northwestern University, Chicago, IL 60611, USA

***Correspondence**: Michael C. Jewett, Department of Chemical and Biological Engineering, Northwestern University, Evanston, IL 60208, USA

E-mail addresses: m-jewett@northwestern.edu

Tel: 1 847 467 5007; **Fax:** 1 847 491 3728

Table S1. Doubling time for strains used in this study grown at different culture volumes.

Strain	Working Volume (L)	Type (L)[a]	T_d (min)[b]	Rate (%)
BL21 Star™ (DE3)	10	Fermenter (15)	32.0	95
	1	Flask (2.5)	30.3	100
	0.5	Flask (2.5)	31.7	96
	0.1	Flask (0.3)	30.8	98
	0.05	Flask (0.3)	34.5	88
	0.01	Test tube (0.02)	54.0	56
K12 MG1655 C495	1	Flask (2.5)	40.8	100
	0.1	Flask (0.3)	40.9	100
	0.01	Test tube (0.02)	52.0	79

(a) Total reactor volume

(b) Doubling time

Rate is given in percentage as compared to 1L working volume shake flask growth.

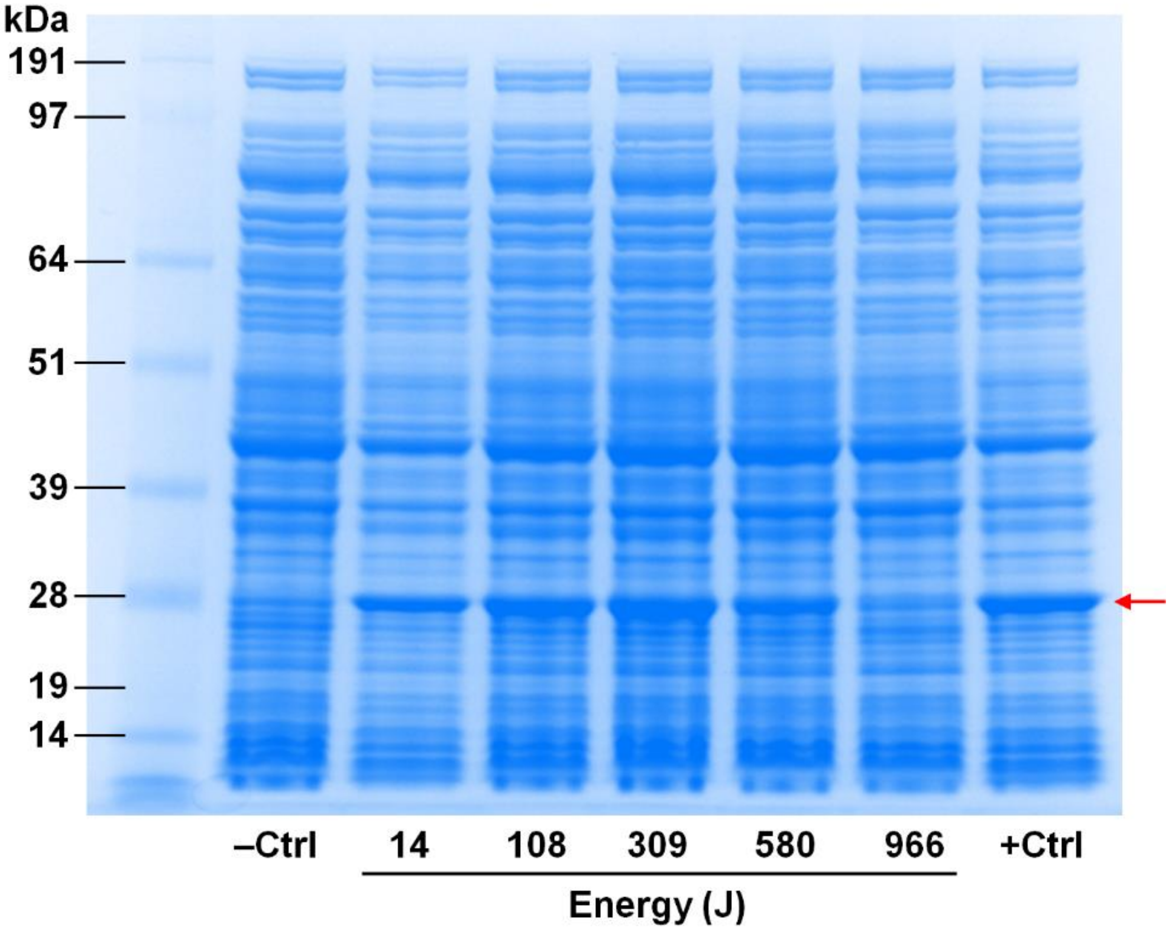

Figure S1. SDS-PAGE analysis for cell-free synthesized sfGFP using crude extracts prepared by sonication at different energy inputs per 0.5 mL cell suspension volume. Red arrow indicates synthesized sfGFP protein. –Ctrl: CFPS in sonicated lysate without sfGFP template DNA lacks the sfGFP protein band. +Ctrl: CFPS of sfGFP in a control extract prepared from 10 L fermentation and cell lysis by impinge homogenization.

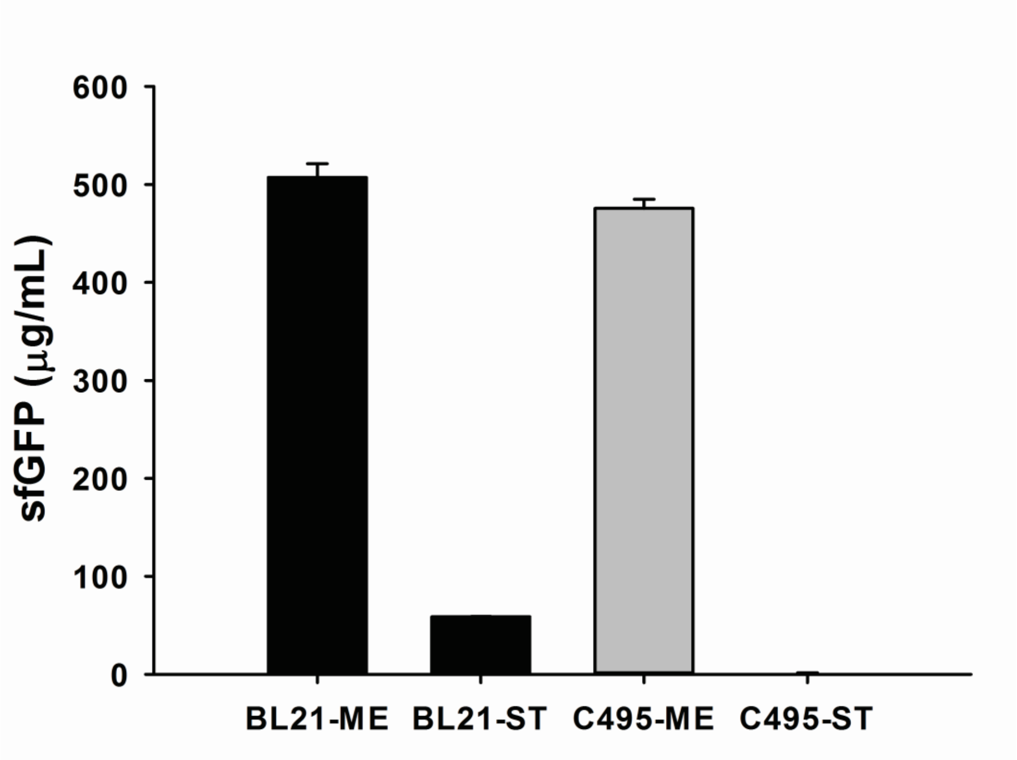

Figure S2. CFPS activity of extracts prepared from mid-exponential (ME) and stationary (ST) phase cells. Optical densities of stationary phase cells were 10 and 8 for BL21 Star™ (DE3) and C495, respectively. Cells were harvested at OD_{600} 3 for mid-exponential phase cells. Black bars are BL21 Star™ (DE3) and gray bars are C495. Active sfGFP synthesis is shown over the course of a standard batch reaction incubated for 4 h. Values represent averages and error bars represent standard deviation for 3 independent experiments.

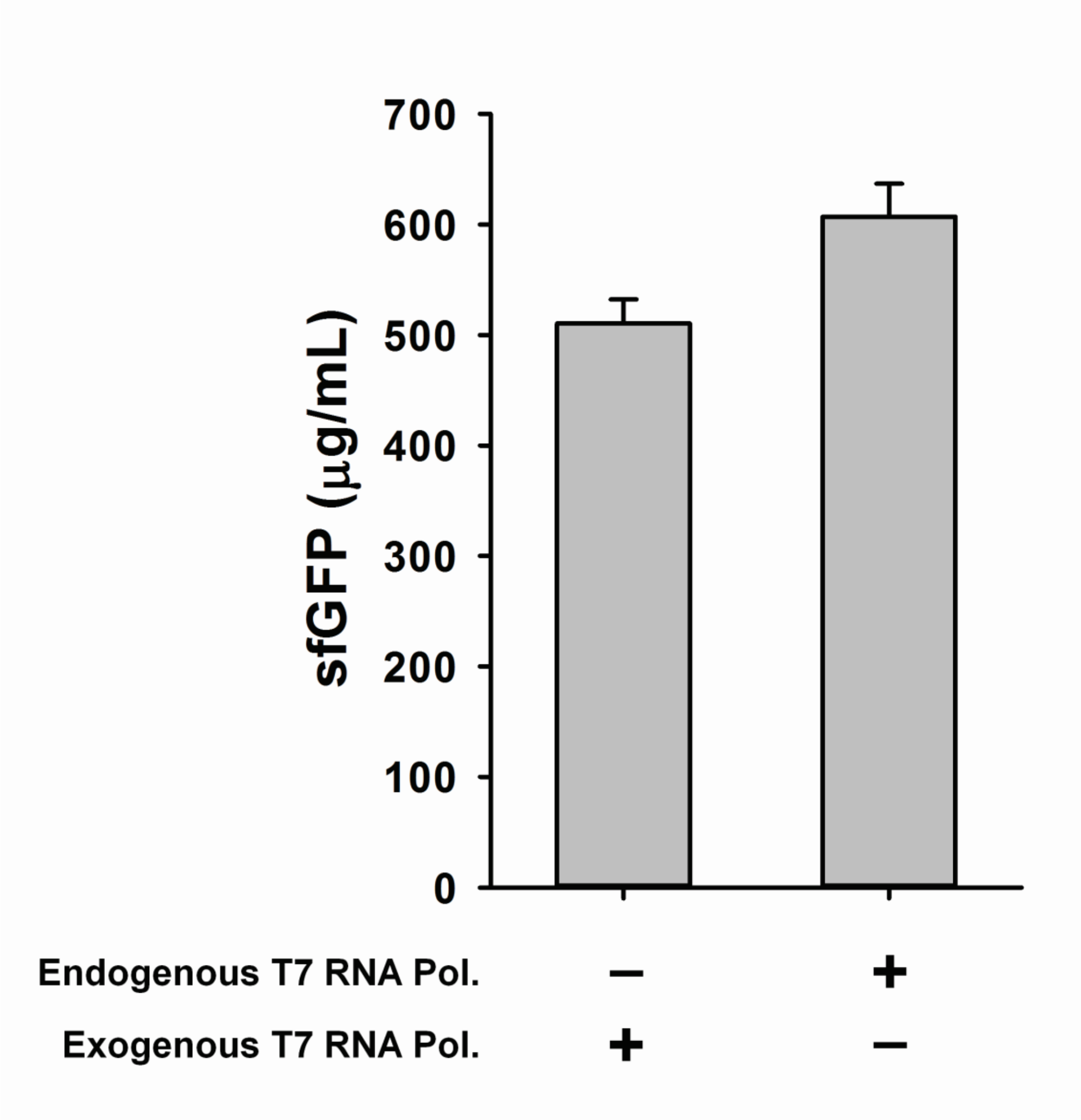

Figure S3. Comparison of CFPS activity in extracts from BL21 Star^{TM} (DE3) cells with or without overexpression of T7 RNA polymerase in the source strain. T7 RNA polymerase was induced at OD_{600} 0.5 by 1 mM IPTG addition and harvested at mid-exponential phase. When T7 RNA polymerase was not overexpressed in the source strain prior to lysis, it was added exogenously (as was done in all other manuscript figures). Detailed cell extract preparation procedure was described in Methods. Active sfGFP synthesis is shown over the course of a standard batch reaction incubated for 4 h. Values represent averages and error bars represent standard deviation for 3 independent experiments.

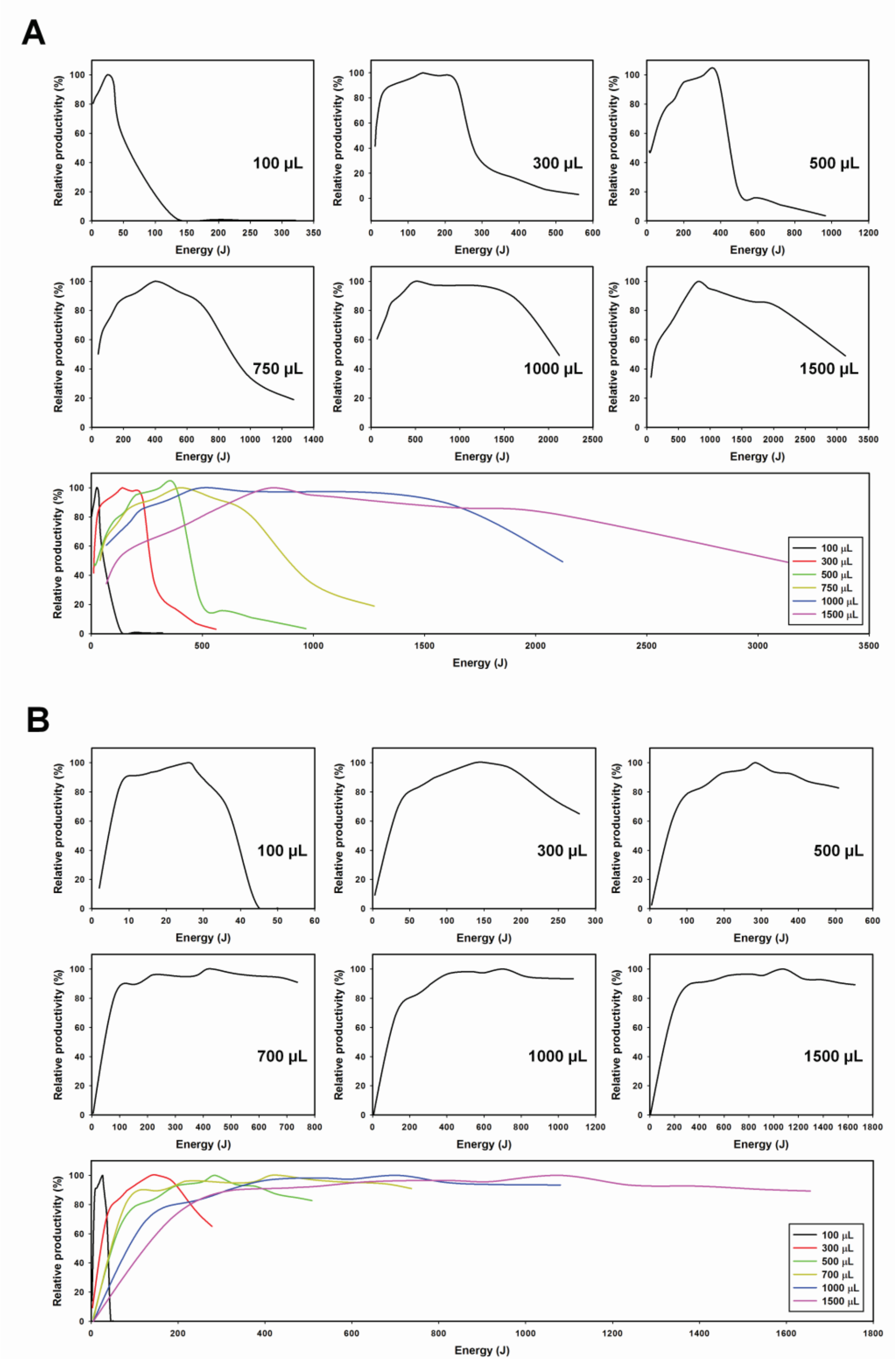

Figure S4. Relative CFPS activities resulting from lysates prepared using different sonication energy input for different cell suspension volumes. All extracts were prepared using 10s sonication - 10s cooling intervals. **A)** BL21 Star^{TM} (DE3); Bottom, combined data of upper 6 small figures. **B)** C495; Bottom, combined data of upper 6 small figures.

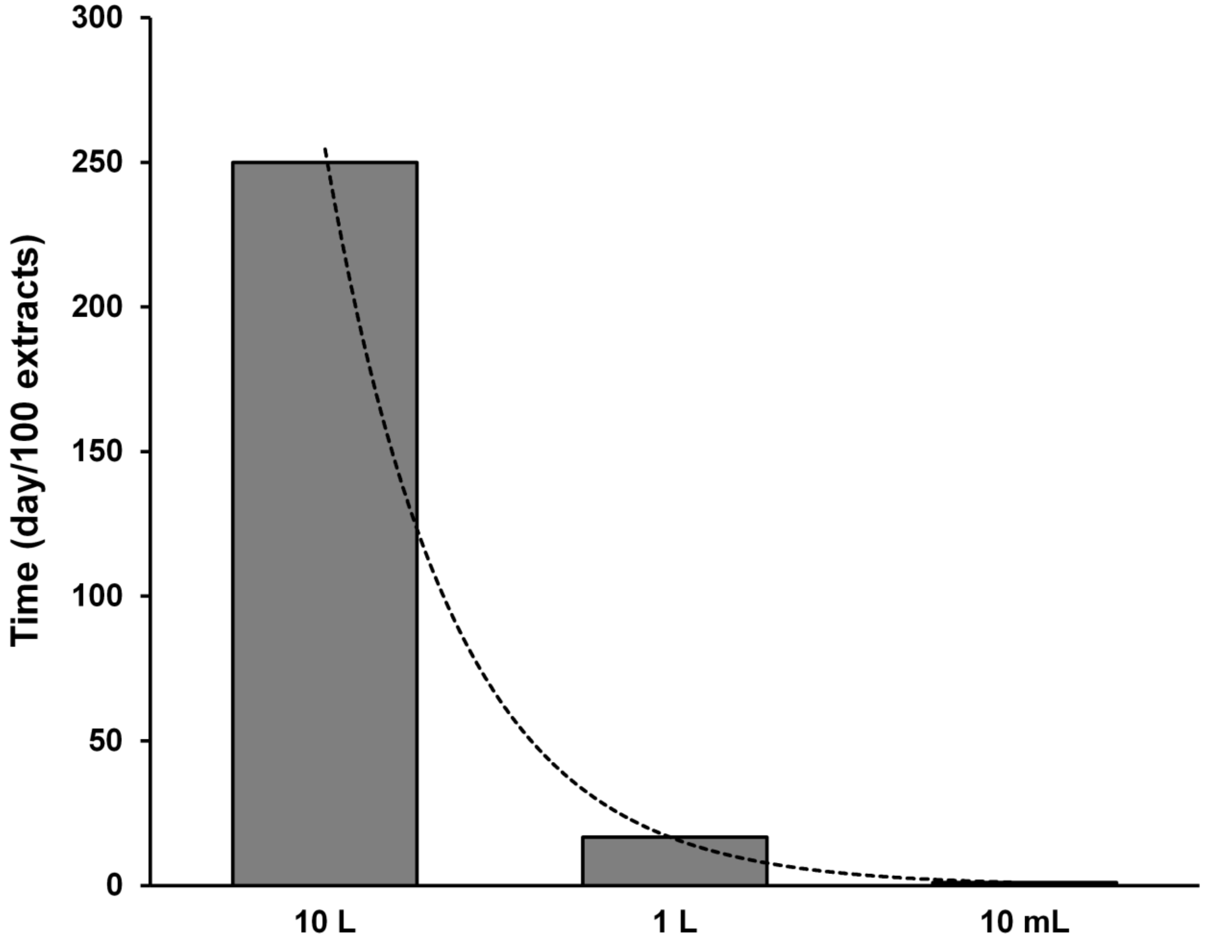

Figure S5. Time reduction for the preparation of highly active crude extracts resulting from the sonication method described in this work. The comparison of days to make 100 extracts for one person using a 10 L (fermenter), 1 L (shake flask) and 10 mL (culture tube) culture is shown.

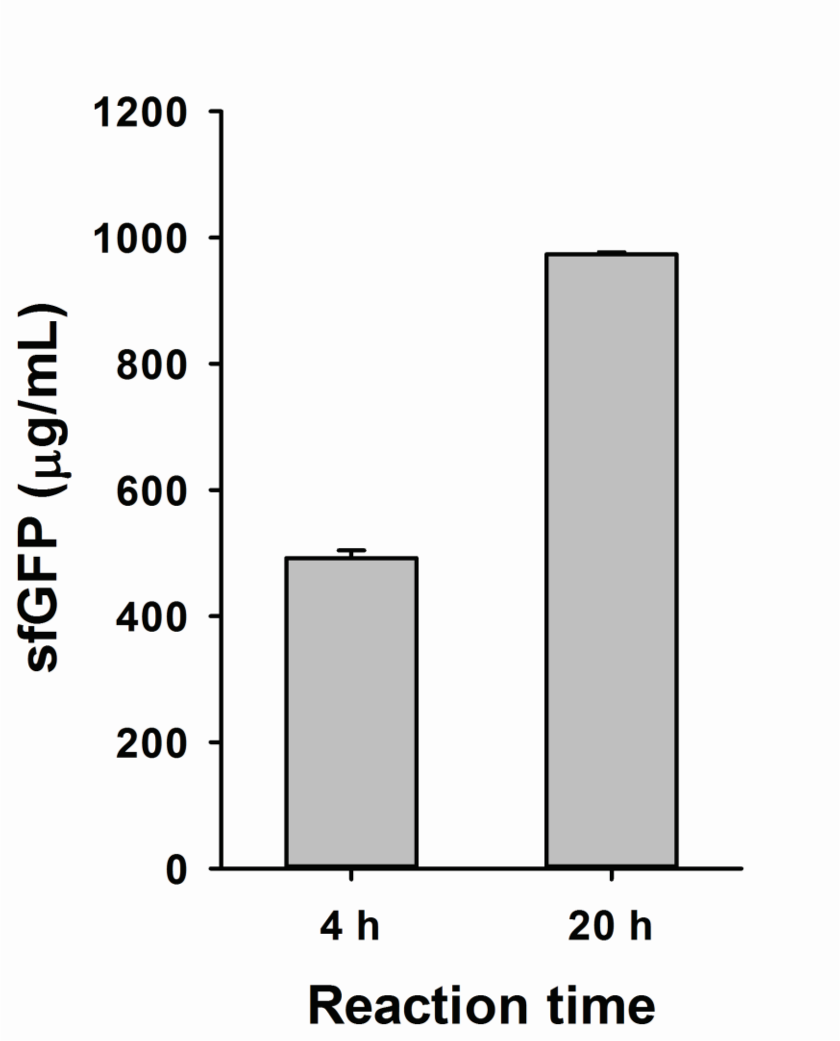

Figure S6. CFPS expression yields are increased when batch reactions are allowed to go to completion. Active sfGFP synthesis is shown over the course of a standard batch reaction incubated for either 4 h (as done in the prototyping reactions in the main body of the manuscript) or 20 h (after the termination of the batch reaction). Fifteen μL batch reactions were prepared in separate 1.5 mL tubes and sampled for active sfGFP yield at the appropriate time points and measured using fluorescence intensity. Values represent averages and error bars represent standard deviation for at least 3 independent experiments.
